# Type and Location of Adenomyosis in Women with Recurrent Pregnancy Loss: A Transvaginal Ultrasonographic Assessment

**DOI:** 10.1007/s43032-024-01541-8

**Published:** 2024-04-15

**Authors:** Caterina Exacoustos, Carlo Ticconi, Irene Colombi, Giuseppe Gabriele Iorio, Elena Vaquero, Aikaterini Selntigia, Barbara Chiaramonte, Giorgia Soreca, Giuseppe Rizzo

**Affiliations:** 1 Department of Surgical Sciences, Obstetrics and Gynecological Unit, University of Rome ‘Tor Vergata’, Rome, Italy; 2 Department of Molecular and Developmental Medicine, Obstetrics and Gynecological Clinic University of Siena, Siena, Italy; 3 Department of Neuroscience, Reproductive Science and Odontostomatology, University of Naples ‘Federico II’, Naples, Italy; 4 Department of Biomedicine and Prevention, Obstetrics and Gynecological Unit, University of Rome ‘Tor Vergata’, Rome, Italy

**Keywords:** Adenomyosis, Ultrasound, Recurrent Pregnancy Loss, Uterus, Endometriosis

## Abstract

The current knowledge on adenomyosis as a risk factor for RPL is very scant. Overall 120 women were included in this retrospective observational study. They were divided in three groups each of which consisted of 40 subjects: Group 1: women with RPL who were diagnosed to have adenomyosis on transvaginal ultrasound (TVS); Group 2: patients with RPL without ultrasonographic findings of adenomyosis; Group 3: patients with ultrasound diagnosis of adenomyosis without RPL and at least one live birth pregnancy. The copresence of endometriosis was also investigated. Among women with RPL, patients with adenomyosis (Group 1) had higher number of pregnancy losses (*p* = 0.03) and lower age at first pregnancy loss (*p* = 0.03) than women without adenomyosis (Group 2). Moreover, they had more frequently primary RPL (*p* = 0.008). Adenomyosis of the inner myometrium was found more frequently (*p* = 0.04) in patients of Group 1 than in patients of Group 3 in which adenomyosis was mainly in the outer myometrium (*p*= 0.02). No differences were found in the severity of adenomyosis between these two groups of women. TVS findings for endometriosis were observed more frequently in women with adenomyosis without RPL (Group 3) than in the other two groups of patients. Adenomyosis can be a factor involved in RPL. Differences in adenomyosis localization are associated with different risks for RPL. Patients with RPL should be investigated for the presence of adenomyosis and also for the type and localization of the disease in the different myometrial layers.

## Introduction

Recurrent pregnancy loss (RPL) defined, according to the European Society of Human Reproduction and Embryology (ESHRE), as the loss of two or more pregnancies before 24 weeks of gestation [1], is a still enigmatic condition. In fact, even though there is a general consensus that RPL is a multifactorial condition [2] , at present the risk factors for RPL remain largely undetermined, so that in only around 50% of couples with RPL a risk factor or cause can be found [3]. Therefore, there is the urgent need to determine the potential involvement in RPL of still poorly investigated factors or conditions. This is the case of adenomyosis, defined as the presence of ectopic endometrium, endometrial glands and stroma developing in the myometrium [4–6] Indeed, the recently updated ESHRE guidelines on recurrent pregnancy loss for the first time recommend US evaluation for adenomyosis in patients with RPL [1].

To date, most studies focused their interest on the relationship between adenomyosis and infertility [7] or on the outcome of women with adenomyosis undergoing assisted reproductive technologies [8–11]. However, the experimental evidence on adenomyosis and RPL is still scant; to our knowledge there is only one study carried out on this issue in non-infertile women [12].

The major problem related to adenomyosis is the diagnosis in fertile patients where histology of uterine specimens is not possible. High-resolution transvaginal ultrasound (TVS) has improved the diagnosis of adenomyosis and is actually the first-line noninvasive diagnostic tool in the detection of adenomyosis [13–17]. The recent interest to this noninvasive TVS diagnosis has led to several attempts to describe ultrasonographic signs of the pathology and to classify the disease [18–20]. The Morphological Uterus Sonographic Assessment (MUSA) consensus proposed 8 sonographic criteria to recognize adenomyosis in US evaluation [14–16]. Some of these criteria are direct signs of the disease in the myometrium whereas others are indirect features induced by the inflammation and fibrosis due to the activity of endometrial glands inside the myometrium. Although no consensus has been reached yet on the classification, authors agree that adenomyotic lesions, visualized by TVS, should be accurately described, reporting in detail: 1) the involvement of one or more uterine walls; 2) the type (diffuse, focal, or adenomyomas); 3) the exact localization in one or more myometrial layers [16, 17]. Different localizations and types of adenomyosis evaluated by imaging may be correlated to different pathogenetic mechanisms such as infiltration from the endometrium, invasion from endometriosis of the posterior compartment or de novo development of lesions not connected with the endometrium or the serosa [21, 22].

Adenomyosis may alter the myometrial layer and affect its functions, especially uterine peristalsis, determining adverse reproductive outcomes [5, 23]). Several authors believe that the subendometrial layer of the myometrium (junctional zone, JZ) plays a role in reproductive outcome by its involvement in spermatozoa transport as well as in blastocyst transport and implantation [7, 24].

Since adenomyosis can exist in several heterogeneous subtypes and the affected patients have different reproductive outcomes, it is reasonable to assume that the localization of the disease in different myometrial layers and the presence of focal or diffuse lesions exposes patients to different reproductive risks, including RPL. The aim of this study was to investigate the potential or possible impact of adenomyosis on RPL and to correlate different types and localizations of adenomyosis with different reproductive outcomes according to the presence or the absence of RPL.

## Materials and Methods

### Setting and participants

This case-control study was carried out on 120 women attending the Department of Gynecology, Ultrasound Unit and Service of Recurrent Pregnancy Loss, of the Policlinico Tor Vergata University Hospital from January 2018 to February 2023. The study women (age range: 18-45 years) were divided into three Groups:

- Group 1 (*n* = 40): women with RPL and ultrasonographic findings of adenomyosis according to MUSA criteria [14–16] diagnosed before pregnancy or no later than 12 months after the last pregnancy loss;
- Group 2 (*n* = 40): women with RPL without ultrasonographic findings of adenomyosis;
- Group 3 (*n* = 40): women without RPL, with at least one pregnancy ended in live birth and with ultrasonographic findings of adenomyosis. Group 3 patients were diagnosed to have adenomyosis before becoming pregnant.

The three groups were matched 1:1 according to age and BMI, in the same study period. All patients were assessed clinically and by ultrasound. Exclusion criteria were: postmenopausal women, ongoing pregnancy, patients with previous or concomitant malignancy of the genital tract, patients unable to adhere to study procedures or non-consenting patients, and patients with incomplete anamnestic, clinical and ultrasound data.

The present study was carried out in accordance with the Declaration of Helsinki, modified Tokyo 2004, and was approved by the Institutional Review Board (IRB) of Policlinico Tor Vergata University Hospital (protocol number: #73/23). All the included patients had a complete clinical history and signed a consent form for US examination and personal data analysis.

### Clinical Evaluation

For each patient the following data were collected: age, body mass index (BMI Kg/m^2^), menstrual cycle characteristics, last menstrual period, parity (number of all previous pregnancies: spontaneous abortions and/or live births), modality of delivery, type of infertility, previous surgery, any other medical or surgical disease.

Ongoing medications were registered, including hormone and non-steroidal anti-inflammatory drugs, replacement therapies for thyroid disfunctions and anticoagulants.

For each pregnancy loss information was collected about maternal age, date of the last menstrual period, serial beta-HCG levels, presence of embryo’s cardiac activity, gestational age at the end of the pregnancy, histology of abortive tissue, possible subsequent instrumental dilatation and curettage.

Women with RPL were investigated according to a standardized diagnostic workup already reported in detail [25–27]. Briefly, the following items were investigated: uterine anatomical abnormalities, thrombophilias, endocrine and autoimmune disorders, parental karyotype abnormalities, clinical risk factors including maternal age, BMI, cigarette smoking and number of previous pregnancy losses

RPL was defined according to ESHRE [1]. RPL was defined as unexplained when no identifiable causes could be detected. Primary RPL was defined as the absence of previous viable pregnancy beyond the 24th week of gestation; secondary RPL was defined as RPL occurring in women with at least one previous ongoing pregnancy beyond the 24th week of gestation. Non-visualized pregnancy losses at TVS defined according to Kolte et al. [28] (as pregnancy of unknown location PUL or biochemical pregnancy), were considered in the diagnosis of RPL, since it has been shown that they have a prognostic value [28].

The following were recorded for each live birth pregnancy: maternal age at the conception, use of assisted reproductive techniques (ART), gestational age at the delivery, mode of delivery, preterm birth (delivery at <37 weeks of gestation, PTB), placental abnormalities (previa, abruption or retention), hypertensive disorders of pregnancy, gestational diabetes mellitus (GDM) and small for gestational age (SGA, birthweight <10^th^ percentile).

### Ultrasonographic evaluation

According to the ESHRE guidelines for RPL, ultrasound assessment of the uterine anatomy was performed [1]. All the ultrasound examinations were carried out using a Voluson E6 device (GE Healthcare, Zipf, Austria) with a transvaginal probe and were performed by two experienced sonographers (C.E. and C.R.). All patients underwent pelvic evaluation with 2D-ultrasound with greyscale and power Doppler followed by uterus 3D-volume acquisition. All the scans were stored as 2D still images, 2D video clips and 3D volumes. All main pelvic organs and spaces were systematically scanned for the presence of any abnormalities. The sonographer examined uterus, adnexa, pouch of Douglas, bladder, rectum, rectosigmoid junction, ureters, parametrias, rectovaginal septum, vesicouterine pouch, uterosacral ligaments.

The myometrium was carefully evaluated for the presence of direct and indirect ultrasonographic signs of adenomyosis according to MUSA criteria [14–16]. TVS findings like intramyometrial cystic, hyperechoic areas, JZ buds and lines have been identified as ultrasound direct signs of adenomyosis whereas globular uterus, asymmetry and fan shaped shadowing were considered indirect signs of the disease [15]. The diagnosis of adenomyosis was made when at list one direct sign was present.

JZ was evaluated with 3D ultrasound on multiplanar sections and acquired uterine volumes.

For each type of adenomyotic lesion the localization in the inner or outer myometrium was described. In relation to its extension, adenomyosis was divided into focal, diffuse, or adenomyomas and the degree of the disease was calculated according to our previous published classification [17]. Four degrees of extension for each type of disease were considered. A score number from 1 to 4 was assigned for the extension and size of diffuse adenomyosis of the outer myometrium and of the inner myometrium, focal adenomyosis of the inner and outer myometrium and adenomyomas.

The total ultrasound extent of the disease was calculated through the sum of the score numbers obtained and classified as mild, moderate and severe adenomyosis with scores 1-3, 4-6 and ≥7, respectively.

All patients were also evaluated by TVS for the presence of pelvic endometriosis using a previously published US mapping system [29, 30]. The TVS ovarian endometrioma (OMA) diagnosis was defined by the presence of a persistent unilocular or multilocular (<5 locules) cyst characterized by a homogeneous low-level (ground glass) echogenicity of the cyst fluid and absent or moderate vascularization of the cystic walls [31]. The diagnosis of deep infiltrating endometriosis (DIE) was made if at least one structure or organ in the anterior, posterior or lateral pelvic compartment showed the presence of an abnormal retroperitoneal hypoechoic tissue or nodular thickening with irregular contours and no or few Doppler signals according to previously described and validated ultrasonographic criteria [29, 30]. Patients were considered affected by endometriosis if, based on TVS, clear indicative findings were present such as OMA or DIE features. Adhesions without other ultrasound findings of endometriosis were not considered evidence of endometriosis and were considered only if an ovarian endometrioma or typical deep endometriotic lesions were concomitantly detected at the ultrasound scan [29, 30].

The presence of Müllerian uterine malformation was investigated by 3D US and, when present, was classified according to the major classifications [ASRM, ESHRE-ESGE] [32, 33]. Presence, extension and localization of fibroids was described by TVS according to MUSA criteria [14, 16] and FIGO classification system [34].

All sonographic findings of uterine adenomyosis/endometriosis were recorded and stored as images, videos and volumes.

### Statistical Analysis

Statistical analysis was performed using the Statistical Package for the Social Sciences (SPSS v.28.0, IBM).

The determination of sample size was obtained according to the Mathematical Theory of Probability Calculation [35]. In detail: We hypothesized that the three groups of study women (Groups 1, 2 and 3) having the same number of subjects **(*n*** **= 40)** in each Group come from Gaussians populations with known means (**μ**) and unknown variances (**σ**^**2**^); therefore, the respective sample sizes (*n* = 40) are determined, from a mathematical-probabilistic point of view, through the following further hypotheses:

1. We hypothesized the rebuttal of the null hypothesis (| ***μ*** – ***M***_***1,2,3***_| = 0) if the means **μ** of the populations from which the samples are derived (Groups 1, 2 and 3) are different from the corresponding sample averages ***M***_***1***_ ***, M***_***2***_ e ***M***_***3***_ (= ***M***_***1,2,3***_) of a quantity, in absolute value, equal to or greater than the 51.3% limit of the respective standard deviations (**SD**);
2. To verify the rebuttal of the null hypothesis (| ***μ*** – ***M***_***1,2,3***_| = 0) and the alternative hypothesis (| ***μ*** – ***M***_***1,2,3***_| ≠ 0), we used the two-tailed Gauss test z; this test with ***α = 0,05*** **and** ***β = 0,10*** and a 90% [= (1 – β) * 100] power allows, with the above limit (51.3%) and hypotheses, the mathematical-probabilistic determination of the suitability of the aforementioned sample sizes (40 women for each Group).

The distribution of continuous variables was evaluated by the Shapiro test. Continuous variables are expressed as mean ± SD or median and range according to the distribution of the values.

The normally distributed data were analyzed by using two-sided t-tests for independent samples when pairwise comparisons were performed. Multiple comparisons were analyzed by using one way ANOVA followed by Tukey-Kramer test as post hoc test.

The non-normally distributed data were analyzed by using the two-sided Mann-Whitney U-test when pairwise comparisons were performed. Multiple comparisons were analyzed by using Kruskal–Wallis followed by Dunn’s test as post hoc test.

Categorical variables are expressed as percentages and were analyzed by using the Chi-square. Bonferroni’s correction was used as post hoc test for multiple comparisons.

In patients with RPL a multivariate logistic regression analysis was performed. The model assumes adenomyosis as dependent variable and as independent variables: uterine fibroids, TSH >2.5 mU/L, ANA positivity and LA positivity. The presence of disorder was coded as 1 if present and 0 if absent.

## Results

The general and reproductive characteristics of patients included in the study are presented in Table 1. No differences were observed among the study groups with regard to age and BMI. Women with RPL and adenomyosis (Group 1) had significantly higher number of pregnancy losses than both women with RPL but without adenomyosis (Group 2) and women with adenomyosis but without RPL (Group 3). As expected, also women of Group 2 had a higher number of pregnancy losses than women of Group 3.

**Table 1:** General characteristics of study women

	Women with RPL and Adenomyosis (Group 1) [*n*=40]	Women with RPL without Adenomyosis (Group 2) [*n*=40]	Women with adenomysis without RPL (Group 3) [*n*=40]	*p*-value
Age [years] at diagnosis	36.5 (26-45)	36.5 (23-42)	35 (27-40)	K-W: H = 3.65 *P* = 0.16
BMI [Kg/m^2^]	23.28 (19.22-25.14)	22.86 (18.52-25.16)	21.45 (18.9-25.47)	K-W: H = 5.79 *P* = 0.06
Number of pregnancy losses	3.22 ± 1.89*	2.5 ± 0.55**	0.15 ± 0.36***	ANOVA: F= 84.26 ***p*<0.001**
Number of pregnancies	3.55 ± 1.91^§^	2.85 ± 0.86^§§^	1.22 ± 0.48^§§§^	ANOVA: F= 36.99 ***p*<0.001**
Number of Live Births	0 (0-2)	0 (0-2)	1 (1-2)^°^	K-W: H = 72.71 ***p*<0.001**

Parametric continuous data are expressed as mean + SD and were analyzed by using one way ANOVA. Tukey-Kramer test was used for multiple comparisons among groups: *p<0.02 Group1 vs Group 2; ** *p*<0.001 Group 2 vs Group 3; *** *p*<0.001 Group 3 vs Group 1;^§^ *p*<0.03 Group 1 vs Group 2; ^§§^ *p*<0.001 Group 2 vs Group 3; ^§§§^ *p*<0.001 Group 3 vs Group 1

Non-parametric continuous data are expressed as median and range and were analyzed by using Kruskal–Wallis (K-W). Dunn’s test was used for multiple comparisons among groups: ^°^ *p*<0.001 vs Group 1 and vs Group 2

The three groups also differed with regard to the number of pregnancies (*p* <0.001) and live births (*p* <0.001). Patients in Group 1 and Group 2 had a higher number of pregnancies, but fewer live births, than women of Group 3. This finding could be explained by the fact that women with RPL, in order to reach a successful pregnancy, tried to become pregnant more often than women of Group 3.

The characteristics of pregnancy losses (PLs) in women with RPL according to the presence or absence of adenomyosis are reported in Table 2. Women with RPL and adenomyosis (Group 1) had a significantly higher number of PLs than women with RPL but without adenomyosis (Group 2). Moreover, they had a higher number of primary PLs, an overall higher number of losses (>3 PLs) and were younger than women with RPL without adenomyosis. No differences were observed between the two groups of women with regard to secondary RPL. The gestational age at miscarriage and the live birth rate were not influenced by the presence of adenomyosis.

**Table 2:** Characteristics of pregnancy losses (PLs) in women with RPL according to the presence or absence of adenomyosis

Characteristics of Pregnancy loss	Women with RPL		*p*-value
	WITH ADENOMYOSIS (Group 1) [*n*=40]	WITHOUT ADENOMYOSIS (Group 2) [*n*=40]	
Median (range) number of PLs	3 (2-12)	2 (2-4)	**0.03°**
Mean number of primary PLs	3.02 ± 1.93	2.17 ± 1.01	**0.008**°°
Mean number of secondary PLs	0.25 ± 0.81	0.32 ± 0.83	0.34°
Number of patients with >3 PLs	24 (60%)	19 (47.5%)	0.26**°°°**
Mean age (years) at first PL	31.82 ± 5.45	34.1 ± 3.97	**0.03**°°
Median (range) gestational age at PL (weeks)	8 (5-16)	8 (4-17)	0.20°
Live Births	5 (12.5%)	6 (15%)	0.74°

°Mann-Whitney two-sided U-test; °° t-tests for independent samples; °°° Chi-square test

The distribution of several potential contributing factors to RPL (TSH > 2.50 mU/L, parental karyotype abnormalities, acquired and hereditary thrombophilia, ANA positivity, uterine congenital abnormalities, uterine fibroids and increased resistance of the uterine arteries with PI>2.5) according to the presence or absence of adenomyosis in women with RPL is reported in Table 3. No statistically significant difference was found for any of the factors considered, including specific thrombophilia tests, with the exception of uterine fibroids, that were more frequently present (50% vs 12.5%) in women with adenomyosis. More than half of these fibroids were subserosal, with only one submucous fibroid present in a women belonging to Group 1.

**Table 3:** Distribution of several potential contributing factors to RPL according to the presence of adenomyosis

	Women with RPL		*p*-value (Chi-square test)
	WITH ADENOMYOSIS (Group 1) [*n*=40]	WITHOUT ADENOMYOSIS (Group 2) [*n*=40]	
Possible contributing factors for RPL			
TSH > 2.50 (mU/L)	10 (25%)	12 (30%)	0.62
Parental Karyotype abnomalities	4 (10%)	4 (10%)	1
Acquired thrombophilia^1^	6 (15%)	9 (22.5%)	0.39
• LA	2	6	
• ACA	3	3	
• aβ2GPI	1	0	
Hereditary thrombophilia^2^	26 (65%)	28 (70%)	0.63
• MTHFR mutation	23	25	
• Prothrombin mutation	2	1	
• Factor V Leiden mutation	3	2	
ANA positivity	8 (20%)	11 (27.5%)	0.43
Uterine congenital anomalies	8 (20%)	6 (15%)	0.56
• Dysmorphic uterus (U1)	4	3	
• Septate uterus (U2)	4	3	
Uterine fibroids	20 (50%)	5 (12.5%)	<0.001
• submucosal	1	0	
• intramural	6	2	
• subserosal	13	3	
Increased resistance of uterine arteries (PI >2.5)	10 (25%)	9 (22.5%)	0.79

^1^ At least one positivity to LA, ACA, aβ2GPI

^2^ At least one the following: Factor V Leiden mutation, prothrombin mutation, MTHFR mutation, Protein C, Protein S, Antithrombin deficiency

In order to further explore this finding, a multivariate logistic regression analysis for selected potential predictor factors of recurrent pregnancy loss (uterine fibroids, TSH >2.5 mU/L, ANA positivity and LA positivity) was carried in patients with RPL taking adenomyosis as dependent variable. The results, reported in Table 4, confirmed the positive association between adenomyosis and uterine fibroids in women with RPL (OR = 7.7, 95% CI 2.54-27.5), while no significant association was found for any of the other factors considered.

**Table 4:** Multivariate logistic regression analysis for potential predictor factors of recurrent pregnancy loss taking adenomyosis as dependent variable in patients with RPL

Features	Estimate	Std. Error	t value	*p* value
Uterine fibroids	2.04	0.6	3.41	<0.001
TSH > 2,50 (mU/L)	1	0.54	1.85	0.06
ANA positivity	-0.35	0.99	-0.35	0.72
Acquired thrombophilia (LA positivity)	0.28	0.77	0.36	0.72

The specific characteristics of adenomyosis - localization and severity, classified as previously published [17, 36] - were investigated according to the presence or absence of RPL by comparing women with adenomyosis with and without RPL (Groups 1 and Group 3, respectively). The results are reported in Table 5. Adenomyosis localized only in the inner myometrium was found more frequently in women of Group 1 than in women of Group 3, who conversely had more frequently adenomyosis localized in the outer myometrium. Similar findings were observed with regard to focal adenomyosis, that was observed more frequently in the inner myometrium of Group 1 women compared to Group 3 patients, who conversely had more frequently focal adenomyosis of the outer myometrium. No significant difference between the two groups was found with regard to the prevalence of colocalization of adenomyosis in both the inner and the outer myometrium; nor it was with regard to the prevalence of diffuse localization in the inner and outer myometrium. No differences were found in the severity of adenomyosis between the two groups of women, with similar rates for mild, moderate and severe adenomyosis (Table 5).

**Table 5:** Localization and severity of adenomyosis according to the presence of RPL

	Women with ADENOMYOSIS			
	WITH RPL (Group 1) [*n*=40]	WITHOUT RPL (Group 3) [*n*=40]	*χ*^2^	*p*-value
Localization of Adenomyosis				
Only Inner myometrium	11 (27.5%)	4 (10%)	4.02	0.04
Only Outer myometrium	16 (40%)	26 (65%)	5.01	0.02
Inner and Outer myometrium	13 (32.5%)	10 (25%)	0.55	0.46
Focal of inner myometrium	9 (22.5%)	0 (0%)	7.31	0.001
Focal of outer myometrium	3 (7.5%)	10 (25%)	4.5	0.03
Diffuse of inner myometrium	2 (5%)	4 (10%)	0.72	0.39
Diffuse of outer myometrium	13 (32.5%)	16 (40%)	0.49	0.48
Severity of Adenomyosis				
Mild	31 (77.5%)	28 (70%)	0.58	0.44
Moderate	6 (15%)	9 (22.5%)	0.74	0.39
Severe	3 (7.5%)	3 (7.5%)	0	1

The prevalence and localization of endometriosis among all the study groups were also determined. The results are reported in Table 6. TVS findings for endometriosis were observed more frequently and with statistical significance in women with adenomyosis without RPL (Group 3) than in the other two groups of patients. Indeed, even though the prevalence of endometriosis (30%) in women with RPL and adenomyosis (Group 1) was higher than that (7.5%) found in women with RPL without adenomyosis (Group 2,), this difference became no statistically significant when Bonferroni’s correction (with significance set at *p*<0.0016) was used as post hoc test between these two groups. The higher rates of endometriosis in women of Group 3 was primarily due to the high prevalence (57.5%) of DIE in these patiens. Again, even though the prevalence of DIE (22.5%) in women with RPL and adenomyosis (Group 1) was higher than that (5%) found in women with RPL without adenomyosis (Group 2,), this difference became no statistically significant when Bonferroni’s correction was used as post hoc test between these two groups. No overall significant differences were found in the prevalence of ovarian endometrioma, alone or combined with DIE. In this last instance although an overall difference was observed, however no significant difference was found for any comparison among groups when Bonferroni’s correction was applied to the obtained data (Table 6).

**Table 6:** Prevalence and localization of non-invasive TVS findings for endometriosis among the study groups

	Women with RPL and Adenomyosis (Group 1) [*n*=40]	Women with RPL without Adenomyosis (Group 2) [*n*=40]	Women with Adenomyosis without RPL (Group 3) [*n*=40]	*χ*^2^	*p*-value
Endometriosis	12 (30%)*	3 (7.5%)	31 (77.5%)**, ***	43.21	<0.00001
OMA	3 (7.5%)	0 (0%)	2 (5%)	1.10	0.57
DIE	9 (22.5%)^§^	2 (5%)	23 (57.5%) ^§§^, ^§§§^	28.15	<0.001
DIE+OMA	0 (0%)	1 (2.5%)	6 (15%)	6.69	0.035

Endometriosis = at least one TVS features of endometriosis in the pelvic compartments, OMA = ovarian endometrioma; DIE: deep infiltrating endometriosis

Bonferroni’s correction (significance at *p*<0.0016) was used for multiple comparisons among groups:

- For Endometriosis: * *χ*^2^_= 6.64, *p*=0.0099 (not significant) Group 1 vs Group 2;_ ** *χ*^2^_= 18.15 Group 3 vs Group 1, *P*=0.00002; ***_χ^2^_= 40.10, *p*<0.00001 Group 3 vs Group 2_

- For DIE: ^§^ *χ*^2^_= 5.16, *p*=0.02 (not significant)_ _Group 1 vs Group 2__;_^§§^ *χ*^2^_= 10.20, *p*=0.0013 Group 3 vs Group 1;_^§§§^ *χ*^2^_= 25.65, *p*<0.00001 Group 3 vs Group 2;_

- For DIE+OMA: no significant difference was found for any comparison among groups when Bonferroni’s correction was applied

## Discussion

The potential role of adenomyosis on RPL is still largely unexplored. This is due to two major reasons: a) the interest towards adenomyosis has been mainly focused so far on the impact of this condition on infertility [8–11] rather than on RPL; b) the difficulty in establishing a reliable and shared non-invasive diagnosis of adenomyosis. The recent revised definition of the MUSA classification of ultrasonographic characteristics of adenomyosis [14–16] provides a useful, non-invasive tool to diagnose this disease in fertile women. An important strength of this study is the diagnosis of adenomyosis by a detailed classification of the disease based on a non-invasive tool such as 2D and 3D TVS. This allows to detect the presence of adenomyosis as well as to define the type and the localization of the disease in different layers, so enhancing the possibility to better correlate the type of the disease to different pregnancy outcomes. This diagnostic method therefore allows the study of the relationship between the presence of adenomyosis and RPL an also the assessment of specific characteristics of adenomyosis more likely to be involved in RPL. The recently published ESHRE guidelines on RPL introduced as a new recommendation that all women with RPL could have 2D ultrasound to rule out adenomyosis [1]. This conditional recommendation was based on two studies who however were performed only in the setting of IVF [8, 37].

The present study has been carried out to specifically explore the potential relationship between adenomyosis and RPL. To accomplish this aim we investigated the clinical and ultrasonographic characteristics in three groups of women: 1) women with RPL and adenomyosis; 2) women with RPL without adenomyosis and 3) women with adenomyosis without RPL. This approach, even in a study with a retrospective design, allowed us to suggest several considerations on this issue.

The overall results of our study provide evidence that adenomyosis can be significantly involved in RPL. Indeed, the women with RPL and adenomyosis (Group 1) had higher number of pregnancies and at the same time of pregnancy losses than the other women (Groups 2 and 3, Table 1). This finding could be related to the intriguing relationship between adenomyosis, fertility and RPL. It appears that in women with RPL adenomyosis in itself could not be an obstacle to pregnancy, supporting the overall concept that the endometrium of RPL women is more permissive to low-quality embryos than that of normally fertile women, as suggested by recent evidence [38, 39]; rather, adenomyosis in women with RPL could be a contributing factor in impairing the successive development of an early implanted embryo. This finding is in accordance with and confirms previous observation carried out in patients undergoing oocyte donation [40]. This hypothesis is further supported by the findings obtained when the characteristics of pregnancy losses (PLs) in women with RPL were evaluated according to the presence or absence of adenomyosis (Table 2). Compared with women with RPL without adenomyosis (Group 2), women with RPL and adenomyosis (Group 1) were younger at their first miscarriage and had higher number of PLs. Moreover, their RPL was mainly primary rather than secondary, suggesting that the disease already present in young women could be a relevant factor of their RPL.

There are very few studies specially designed to explore the relationship between adenomyosis and RPL. Atabekoğlu et al., in a prospective controlled study, evaluated their RPL patients for adenomyosis by 2D ultrasound and found an association between RPL and adenomyosis [12]. Our study has been carried out with different research design aimed to specifically investigate the ultrasound features of adenomyosis of particular interest in the setting of RPL; in the present study all patients underwent both 2D and 3D ultrasound; moreover, the diagnostic criteria of adenomyosis were different. Despite these differences, the present study further supports and extends the concept that adenomyosis has a non-negligible impact on RPL. The analysis of the distribution of several factors potentially involved in RPL in our study patients, stratified according to the presence or absence of adenomyosis (Table 3 and Table 4), revealed that the only factor detected more frequently in women with adenomyosis was represented by uterine fibroids. This is not surprising, since a positive association between uterine fibroids and adenomyosis has been described [41]. The relationship between uterine fibroids and RPL is still unclear, due to the lack of prospective, controlled studies specifically designed to explore this issue, with specific application to the actual impact of the site and number of fibroids on RPL. However, the current literature indicates that submucousal fibroids seem to be mainly involved in RPL, while considering intramural and subserous fibroids less relevant in RPL [42, 43]. Accordingly, the position of many international guidelines with respect to this issue is variable [44]. In the present study, we actually found that a significantly higher rate of uterine fibroids was present in Group 1 (women with RPL and adenomyosis) than in Group 2 (women with RPL without adenomyosis) patients, as shown in Table 3. However, nearly all (24/25) fibroids detected in our study in the overall population of RPL women were intramural and/or subserous, i.e. in the supposed uterine location with low-risk/relevance for RPL. The above considerations support the concept that adenomyosis, more than uterine fibroids, is associated with RPL. Moreover, we also found (Table 4) that taking adenomyosis as dependent variable, uterine fibroids were the only significant predictor factor, among those considered, in women with RPL. This finding underlines the need for further studies designed to better disentangle the relative relevance of uterine fibroids and adenomyosis, which often coexist, in the pathogenesis of RPL. Further investigation is needed to fully clarify the significance of this association and the impact of type, number and size of fibroids plus adenomyosis.

The TVS characteristics of adenomyosis were comparatively investigated in women with and without RPL (Table 5). While there were no differences in the severity, however relevant differences were found with regard to the localization of the disease. Women with RPL had significantly higher adenomyosis in inner myometrium and significantly lower adenomyosis in outer myometrium than women with adenomyosis but without RPL. The correlation between involvement of inner myometrium or junctional zone (JZ) and higher miscarriage rate suggests that patients with adenomyosis could have an impaired invasion of the blastocyst in the myometrium. Absent or incomplete remodeling of the JZ can affect uterine peristalsis, alter vascular plasticity of the spiral arteries and activate inflammatory pathways [4, 45]. The junctional zone plays a critical role in sperm transport, implantation, and angiogenesis The invasion of the JZ in women with adenomyosis can alter the endometrial-myometrial interface. Thus, aberrations in the junctional zone may lead to altered endometrial receptivity and defective trophoblast invasion or migration which, in turn, can be the cause of the PL [46]. This concept is further supported by the observation that women with adenomyosis in the outer myometrium had lesser PLs and more live births than women with adenomyosis in the inner myometrium (Table 5). Therefore, localization of the adenomyosis could represent an important predictor of the risk of miscarriage.

The results of the present study are in agreement with previous observations on the reproductive relevance of the JZ. Women with specific features such as infiltration of the JZ seem to have an increased miscarriage rate than those with other features [45, 47]. A multicentric study by Iwasawa et al. using MRI classified adenomyosis into three groups: advanced (adenomyosis involving both JZ and outer myometrium), extrinsic (only outer myometrium), and intrinsic (only JZ). A logistic regression analysis adjusted for age, prior miscarriage, and body mass index showed that the extrinsic group had fewer pregnancy losses (odds ratio 0.06; 95 % confidence interval [CI]: 0.00-0.54, *p* = 0.026) and more live births (odds ratio 6.05; 95 % CI: 1.41-29.65, *p* = 0.018) than the advanced group [48].

As a final finding of the present study, we observed that US signs suggestive/diagnostic for endometriosis were more frequently detected in women with adenomyosis without RPL (Group 3) than in the other study patients (Table 6). Similar finding was observed with regard to deep infiltrating endometriosis (DIE), while no differences were found among all groups with regard to ovarian endometrioma (OMA). These findings are in agreement with the pathogenetic hypothesis [21, 22] that the adenomyosis of the outer myometrium is an infiltration of the uterus from external DIE. Indeed, in presence of DIE and external adenomyosis we observed more live births than PLs.

The issue on whether endometriosis is a risk factor for RPL is debated and still unresolved. A systematic review of pregnancy complications in patients with endometriosis did not find evidence supporting the concept that the disease has a major detrimental effect on pregnancy outcome [49]. Conversely, in accordance with with previous studies [50], a recent Danish nationwide cohort study found an association between endometriosis and RPL [51], raising however some discussion [52]. At present, there is no satisfactory explanation for our finding that Group 3 patients (women with adenomyosis without RPL) have a higher rate of endometriosis than the other two Groups of patients (Table 6). Three tentative explanations are the following: a) since one the one hand it is well known that endometriosis, through still incompletely determined mechanisms, affects fertility and on the other hand emerging evidence [39] indicates that normally there is a clear maternal selection of embryos, it can be hypothesized that only embryos with very high developmental potential can successfully implant in women with endometriosis; therefore, these embryos could have a reduced likelihood to be aborted; b) an other potential explanation could be that Group 3 women actually had a substantially high rate of DIE which is often associated with adenomyosis of the outer myometrium (extrnal adenomyosis) [53] but is not associated with RPL (Table 5); c) it is also possible that different biomolecular and immunologic factors are involved in peritoneal endometriosis and in DIE, leading to different outcomes in terms of RPL. Currently there is very scant, if any, information on the relationship between DIE and RPL. Furthermore Group 3 patients had a mean lesser number of pregnancies than the other two groups (Table 1), so having a reduced likelihood to undergo RPL.

In conclusion, our findings demonstrated that adenomyosis is associated with RPL. Specific signs of adenomyosis as the involvement of the JZ seem to be important in evaluating the risk of RPL. This study strengthens the importance of ultrasound analysis and detailed classification of adenomyosis in patients with RPL. The appropriate classification of adenomyosis based on ultrasound features can offer the physicians an important diagnostic tool in the evaluation of factors potentially involved in RPL.

## Data Availability

Data are available on reasonable request.
